# Diet-Induced Obesity Impairs Outcomes and Induces Multi-Factorial Deficiencies in Effector T Cell Responses Following Anti-CTLA-4 Combinatorial Immunotherapy in Renal Tumor-Bearing Mice

**DOI:** 10.3390/cancers13102295

**Published:** 2021-05-11

**Authors:** William J. Turbitt, Shannon K. Boi, Justin T. Gibson, Rachael M. Orlandella, Lyse A. Norian

**Affiliations:** 1 Department of Nutrition Sciences, University of Alabama at Birmingham (UAB), Birmingham, AL 35233, USA; wturbitt@uab.edu; 2 Department of Cell, Developmental and Integrative Biology, University of Alabama at Birmingham (UAB), Birmingham, AL 35233, USA; 3 Graduate Biomedical Sciences, University of Alabama at Birmingham (UAB), Birmingham, AL 35233, USA; Shannon.Boi@STJUDE.ORG (S.K.B.); jtgibson@uab.edu (J.T.G.); rorlande@uab.edu (R.M.O.); 4 Nutrition Obesity Research Center, University of Alabama at Birmingham (UAB), Birmingham, AL 35233, USA; 5 O’Neal Comprehensive Cancer Center, University of Alabama at Birmingham (UAB), Birmingham, AL 35233, USA

**Keywords:** diet-induced obesity, cancer therapy, immunotherapy, T cells

## Abstract

**Simple Summary:**

Immunotherapy use has become standard for many patients with advanced kidney cancer; unfortunately, <50% of patients experience durable responses. Mounting evidence suggests that modifiable factors, such as diet and obesity, impact immunotherapy outcomes. Obesity, a major U.S. health epidemic, blunts anti-tumor immunity and promotes tumor growth in multiple preclinical models. However, the full biological impact of obesity on the T cell responses needed to achieve positive immunotherapy outcomes remains unclear. Here, we studied the effects of obesity on T cell responses following combinatorial immunotherapy in a mouse model of kidney cancer. We found that obesity is associated with blunted effector T cell responses, resulting in diminished immunotherapy outcomes. This therapy produces sustained T cell responses and robust tumor control in obese-resistant mice fed the same high-fat diet. Finding ways to amplify T cell responses within renal tumors from hosts with obesity will be critical for achieving optimal immunotherapy outcomes.

**Abstract:**

Associations between modifiable factors and the efficacy of cancer immunotherapies remain uncertain. We found previously that diet-induced obesity (DIO) reduces the efficacy of an immunotherapy consisting of adenovirus-encoded TRAIL plus CpG oligonucleotide (AdT/CpG) in mice with renal tumors. To eliminate confounding effects of diet and determine whether outcomes could be improved in DIO mice, we evaluated AdT/CpG combined with anti-CTLA-4 in diet-matched, obese-resistant (OB-RES) versus DIO tumor-bearing mice. Therapy-treated OB-RES mice displayed effective renal tumor control and sustained CD4^+^ and CD8^+^ T cell responses. In contrast, therapy-treated DIO mice exhibited progressive tumor outgrowth and blunted T cell responses, characterized by reduced intratumoral frequencies of IFNγ^+^ CD4^+^ and CD8^+^ T cells. Weak effector T cell responses in therapy-treated DIO mice were accompanied by low intratumoral concentrations of the T cell chemoattractant CCL5, heightened concentrations of pro-tumorigenic GM-CSF, and impaired proliferative capacity of CD44^+^CD8^+^ T cells in tumor-draining lymph nodes. Our findings demonstrate that in lean mice with renal tumors, combining in situ T cell priming upstream of anti-CTLA-4 enhances outcomes versus anti-CTLA-4 alone. However, host obesity is associated with heightened immunotherapy resistance, characterized by multi-factorial deficiencies in effector CD4^+^ and CD8^+^ T cell responses that extend beyond the tumor microenvironment.

## 1. Introduction

Approximately two-thirds of adults in the United States are defined as having either overweight (body mass index [BMI] of 25–29.9 kg/m^2^) or obesity (BMI > 30 kg/m^2^) [1]. Obesity increases the risk of developing thirteen different types of cancers, including renal cell carcinoma (RCC) [2]. Obesity can negatively impact immune function and has been shown to blunt anti-tumor immunity and promote accelerated tumor growth in multiple preclinical models [3,4,5,6,7,8,9,10,11,12]. However, obesity-induced alterations in systemic inflammation, signaling factors, and metabolites may have both beneficial and detrimental effects on T cell anti-tumor immunity [13]. Thus, understanding the effects of obesity on CD8^+^ T cell responses to tumors remains an active area of investigation.

Immune-stimulatory therapies are emerging as the “fourth pillar” of cancer treatment for patients with advanced disease, alongside surgery, radiation, and chemotherapy [14]. Immune checkpoint blockade (ICB) agents, such as those targeting CTLA-4 or programmed death (PD)-1, are designed to improve the ability of CD8^+^ T lymphocytes to maintain effector function and kill tumor targets. Despite demonstrating clinical benefit, typically <50% of cancer patients receiving immunotherapies experience objective, durable responses [15,16] and CTLA-4 targeting as a monotherapy has not proven effective in RCC patients [17]. Multiple factors contribute to the limited efficacy of ICB therapies [18,19] and mounting evidence suggests that modifiable lifestyle factors, such as diet and elevated adiposity, can impact therapeutic outcomes [20,21,22,23,24]. However, the association between obesity and the efficacy of ICB agents remains uncertain [25], especially in the context of advanced RCC [26]. This association likely has differential effects depending on the type of cancer, the tumor-immune landscape, links between obesity and the risk for developing that type of cancer, and/or the sex or ethnicity of the patient. For example, a recent meta-analysis by Xu et al. [27] concluded that the protective effects of obesity were evident for both males and females receiving ICBs when all cancer types were pooled. However, subgroup analysis based on cancer type showed that although most cancer patients with high BMI had better responses to ICB, this was not true for patients with RCC, who trended toward worse outcomes with high BMI (HR = 1.10, CI: 0.50–2.41, *p* = 0.810) [27]. A separate study by Sanchez et al. [28] found no significant association between obesity and ICB outcomes in RCC, although their data trended toward obesity being favorable. More recently, we found that RCC patients with obesity at treatment initiation had worse outcomes following anti (α)-Programmed Cell Death-1 (αPD-1) therapy [9]. With the increased use of combinatorial ICB therapies as standard of care for patients with advanced RCC, additional studies are needed to further elucidate these complex relationships and identify the mechanisms that shape outcomes. Furthermore, the full biological impact of obesity on the CD4^+^ and CD8^+^ T cell responses necessary to drive ICB efficacy remains unknown [13].

Here, we sought to better understand the effects of obesity on CD8^+^ T cell responses to renal tumors following administration of a novel combinatorial immunotherapy comprised of in situ T cell priming plus αCTLA-4. T cell priming was accomplished through the intrarenal co-administration of adenovirus encoding murine TRAIL (AdT) plus the immune-stimulatory oligonucleotide CpG (AdT/CpG) in mice with established orthotopic renal tumors. We and others have shown that intratumoral AdT/CpG is effective in lean, but not DIO mice, that have established orthotopic Renca renal tumors [3,29], and αCTLA-4 monotherapy has shown efficacy in lean, but not obese, mice with subcutaneous Renca tumors [30]. Thus, we combined AdT/CpG with αCTLA-4 to evaluate the efficacy of this combinatorial approach in mice with established, orthotopic renal tumors in the presence or absence of host obesity. We found that in lean mice on a low-fat diet, this combinatorial therapy was more efficacious than αCTLA-4 alone. We then examined the effects of host obesity on tumor and immune responses to AdT/CpG/αCTLA-4 combinatorial therapy. To negate potential confounding effects of diet, cohorts of mice were maintained on high-fat diet (HFD) and we compared immune responses and tumor outcomes between subsets of diet-induced obese (DIO) mice and our previously described obese-resistant (OB-RES) mice [31]. We find that AdT/CpG/αCTLA-4 combinatorial therapy is efficacious in lean mice, regardless of diet composition. In contrast, DIO mice experience progressive renal tumor growth, characterized by multi-factorial deficiencies in effector CD4^+^ and CD8^+^ T cell responses after immunotherapy administration. Our data suggest that the negative effects of host obesity on the quality and duration of immunotherapy-induced T cell immunity extend beyond the tumor microenvironment, warranting further investigation of these findings in other systems.

## 2. Materials and Methods

### 2.1. Animals and Diets

Female BALB/c mice were purchased from the NCI-Frederick colony maintained by Charles River Laboratories (Wilmington, MA, USA) at seven to eight weeks of age. Mice were acclimated in-house for one week and fed a standard low-fat chow diet (NIH-31; LabDiet; St. Louis, MO, USA). Mice were then randomized to either remain on standard chow diet or switched to 20 weeks of ad libitum high-fat diet (HFD, catalog #12492; Research Diets; New Brunswick, NJ, USA) feeding to generate obese-resistant (OB-RES) and diet-induced obese (DIO) mice, as previously described [3,31]. All mice were housed in standard caging (*n* = 5/group) under pathogen-free conditions in 12:12 light:dark cycles at 22 °C (72 °F average). All animal procedures were approved by the Institutional Animal Care and Use Committee (IACUC) at the University of Alabama at Birmingham, an AAALAC-accredited institution, on 1 March 2018 (IACUC-21147).

### 2.2. Murine In Vivo Tumor Modeling

The Renca cell line (syngeneic to BALB/c mice) was purchased from the American Type Culture Collection (ATCC; Manassas, VA, USA), engineered to express firefly luciferase, and cultured as described [4,29,32]. Cells were confirmed negative for mycoplasma, passaged, and used at the same passage number to limit experimental variation. Intrarenal tumor challenges and bioluminescent imaging (BLI) using an IVIS Lumina III Imager (Perkin Elmer; Waltham, MA, USA; University of Alabama at Birmingham (UAB) Small Animal Imaging Facility) were performed as described [4,29,32]. At day six post-tumor challenge, BLI was used to assess tumor burdens in live mice prior to therapy randomization. At day seven post-tumor challenge, mice were re-injected in the tumor-bearing kidney with either sterile saline or 10^9^ pfu of replication-deficient adenovirus (Ad) encoding a membrane-bound version of full-length murine TRAIL protein (University of Iowa Viral Vector Core) plus 100 µg CpG1826 (Integrated DNA Technologies) (AdT/CpG treatment) and further randomized to receive no therapy, *InVivo*MAb polyclonal Armenian hamster IgG control (BioXCell; Lebanon, NH, USA), or *InVivo*MAb anti (α)-mouse CTLA-4 (clone UC10-4F10-11; BioXCell) i.p. at a dose of 100 μg/mouse on days 10, 13, and 16 post-tumor challenge.

### 2.3. NanoString Immunogenetic Profiling

RNA was isolated from whole tumors harvested at day 21 post-tumor challenge from no therapy and therapy-treated OB-RES and DIO mice (*n* = 3–8/group). RNA concentration was quantitated using a Take3 micro-volume plate in conjunction with a Synergy reader (BioTek; Winooski, VT, USA). Samples were processed using the nanoString Mouse PanCancer Immune Profiling Kit (nanoString Technologies Inc.; Seattle, WA, USA). Data analyses were performed using nSolver™ Analysis Software (nanoString Technologies, Inc.), by comparing therapy-treated groups to their respective no therapy controls. Due to the exploratory nature of this study, an unadjusted *p* value less than 0.05 was used to screen for differentially expressed genes. Differentially expressed T cell related genes (listed in Supplemental Table S1) were queried using the Reactome Database [33].

### 2.4. Flow Cytometry

Renal tumors were homogenized and processed as described to yield single-cell suspensions [4,29,32]. Tumor-draining lymph nodes (TDLNs) from tumor-bearing mice were harvested and single-cell suspensions were generated by disruption with glass slides. Cells were stained with Zombie Aqua Fixable Viability Dye (Biolegend; San Diego, CA, USA) followed by TruStain FcX (Biolegend) to block Fc receptors. Cells were then extracellularly stained with saturating concentrations of conjugated antibodies. For IFNγ intracellular staining, cells were ex vivo stimulated with purified αCD3 and αCD28 (BioLegend) for four hours. GolgiPlug protein transport inhibitor (BD Biosciences; San Jose, CA, USA) was added for the final two hours. Cells were harvested and extracellularly and intracellularly stained using BD Biosciences Fixation/Permeabilization Solution Kit. Annexin V staining was done in accordance with the BD Annexin V apoptosis detection kit protocol. Results were obtained from multi-parameter flow cytometry using an Attune NxT Flow Cytometer (ThermoFisher Scientific; Waltham, MA, USA) or BD LSR II (BD Biosciences) and analyzed with FlowJo software (BD Biosciences). Doublets were excluded by FSC-A/FSC-W gating, and dead cells were excluded via Zombie Dye. Positive events were objectively determined using fluorescence minus one (FMO) controls.

### 2.5. Tumor-Specific Protein Quantification

Tumors were isolated, normalized to 0.2 g/1 mL DPBS, and homogenized. Tumor supernatant was collected and stored at −80 °C. Tumor supernatants were thawed on ice, then soluble cytokines and chemokines were quantified according to the manufacturer’s instructions using the Bio-Plex Pro Mouse Chemokine 31-plex (Bio-Rad; Hercules, CA, USA). Samples were run in duplicate on a MagPix instrument (Luminex; Austin, TX, USA) according to the manufacturer’s protocol.

### 2.6. Statistical Analysis

All data were assessed for normality (Shapiro–Wilk normality test) and equal variances, and either parametric or non-parametric analyses were used to detect differences between treatment groups. For studies involving two groups, unpaired Student’s *t*-tests or non-parametric Mann–Whitney U-tests were performed as appropriate. For three or more groups, one-way ANOVA or non-parametric Kruskal–Wallis tests were performed as appropriate. Pairwise comparisons between groups of interest using Dunn’s post hoc tests were performed to correct for multiple comparisons. For experiments examining repeated measures over time between two or more groups, two-way ANOVAs were performed with repeated measures or mixed models design as necessary, followed by post hoc multiple comparison tests (Bonferroni for two groups, Dunnett’s for more than two). All statistical analyses were performed using Prism 8 (GraphPad Software; La Jolla, CA, USA). All data (from *n* = 2–5 independent experiments) are presented as the mean ± SEM. Asterisks designate significance using parametric testing (* *p* < 0.05, ** *p* < 0.01, *** *p* < 0.001, **** *p* < 0.0001), whereas pound signs designate significance using non-parametric testing (^#^ *p* < 0.05, ^##^ *p* < 0.01, ^###^ *p* < 0.001, ^####^ *p* < 0.0001). Non-significant trending *p*-values (*p* < 0.100) are indicated.

## 3. Results

### 3.1. Combinatorial Therapy Induces the Greatest Reduction in Tumor Growth and Expansion in CD44^+^CD8^+^ Tumor-Infiltrating Lymphocytes (TILs) in Young, Chow-Fed Mice

**Figure 1:**
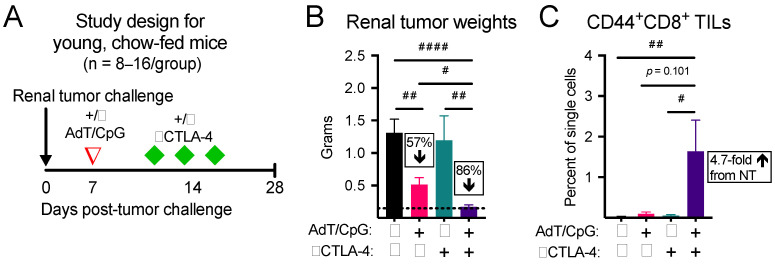
Combinatorial therapy induces the greatest reduction in tumor growth and expansion of CD44^+^CD8^+^ tumor-infiltrating lymphocytes (TILs) in young, chow-fed mice. (**A**) Experimental design. (**B**) Tumor weights and (**C**) CD44^+^CD8^+^ TILs at day 28 post-tumor challenge. Percent change compared to no therapy control. ^#^ denotes non-parametric test. ^#^ *p* < 0.05, ^##^ *p* < 0.01, ^####^ *p* < 0.0001. NT: no therapy.

Anti(α)-CTLA-4 monotherapy has not been FDA approved for use in advanced renal cancer patients, due to a lack of demonstrated efficacy [34]. Here, we began by asking whether in situ T cell priming upstream of αCTLA-4 would control tumor growth and increase the frequency of activated CD8^+^ TILs in tumor-bearing kidneys. Young mice (seven to eight weeks old) with established orthotopic renal tumors were randomized to receive no therapy, AdT/CpG alone, αCTLA-4 alone, or AdT/CpG+αCTLA-4 (Figure 1A). Tumor weights at day 28 post-tumor challenge were significantly different between groups, with the combinatorial AdT/CpG+αCTLA-4 group displaying a significant reduction compared to no therapy and monotherapy groups (Figure 1B, *n* = 8–16/group; Kruskal–Wallis test, KW = 21.43, *p* < 0.001). Furthermore, combinatorial therapy expanded CD44^+^CD8^+^ TILs compared to no therapy and monotherapy groups (Figure 1C, *n* = 4–6/group; Kruskal–Wallis test, KW = 11.27, *p* = 0.010). These results illustrate that our combinatorial approach was suitable for further studies aimed at investigating the effects of obesity on tumor and immune responses to therapy.

### 3.2. Therapy-Treated OB-RES Mice Have a Sustained Reduction in Tumor Growth over Time; Therapy-Treated DIO Mice Experience an Acute, Transient Response Followed by Tumor Outgrowth

**Figure 2:**
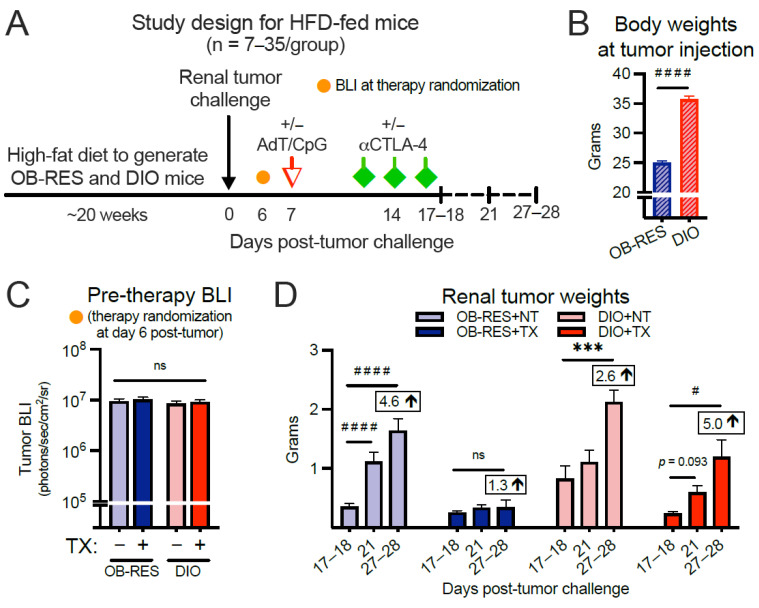
Therapy-treated obese-resistant (OB-RES) mice have a sustained reduction in tumor growth over time, whereas therapy-treated diet-induced obese (DIO) mice experience an acute response followed by tumor outgrowth. (**A**) Experimental design. (**B**) Body weights prior to tumor injection. (**C**) Primary tumor bioluminescence (BLI) prior to therapy administration. (**D**) Tumor growth over time within each treatment group. Data from n = 2–5 independent experiments per time point. Fold change values at day 27–28 compared to day 17–18 value. * denotes parametric and ^#^ denotes non-parametric test. ^#^ *p* < 0.05, *** *p* < 0.001, ^####^ *p* < 0.0001. ns: non-significant, NT: no therapy, TX: AdT/CpG+αCTLA-4.

We proceeded by asking whether obesity would positively or negatively impact renal tumor burdens after combinatorial AdT/CpG+αCTLA-4 therapy. Here, young mice (seven to eight weeks old) were placed on HFD for 20 weeks (Figure 2A) to generate OB-RES and DIO mice, as we described previously [3,31]. Because all mice were diet-matched, this negated the potential impact of dietary changes on any differential outcomes that might be observed between lean and DIO mice. As expected, body weights prior to tumor injection were significantly different between OB-RES and DIO groups (Figure 2B, Mann–Whitney test, *p* < 0.001). DIO and OB-RES mice were injected orthotopically with Renca cells and primary tumor bioluminescence (BLI) was not significantly different between groups prior to the administration of therapy at day six post-tumor challenge (Figure 2C, Kruskal–Wallis test, KW = 1.48, *p* = 0.688). However, progressive tumor growth over time (i.e., days 27–28 significantly different than days 17–18) was observed in OB-RES no therapy (*p* < 0.001) and DIO no therapy (*p* = 0.001) controls, as well as therapy-treated DIO mice (*p* = 0.015), whereas therapy-treated OB-RES mice displayed a sustained reduction (*p* = 0.519) in tumor growth through day 27–28 (Figure 2D). A similar pattern emerged when visualizing the same tumor data by day of sacrifice (Supplemental Figure S1A); significant differences were observed at days 17–18 (Kruskal–Wallis test, KW = 9.38, *p* = 0.025), day 21 (Kruskal–Wallis test, KW = 38.38, *p* < 0.001), and days 27–28 (Kruskal–Wallis test, KW = 24.76, *p* < 0.001) post-tumor implantation, with therapy-treated OB-RES mice displaying a sustained percent reduction in tumor weight compared to no therapy controls. Thus, host obesity impaired the efficacy of this AdT/CpG+αCTLA-4 combinatorial therapy, which was able to control renal tumor outgrowth in both HFD-fed OB-RES mice (Figure 2) and chow-fed lean mice (Figure 1).

### 3.3. Therapy-Treated OB-RES Mice Display an Immunogenetic Profile Characterized by Increased Expression of T Cell Related Genes and Biological Processes

**Figure 3:**
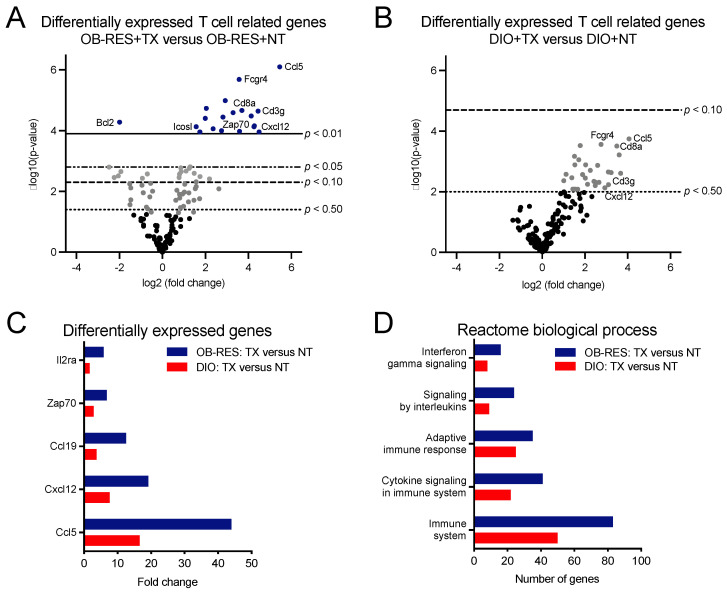
Immunogenetic profiling of renal tumors illustrates that OB-RES mice have a more robust anti-tumor T cell response following therapy administration than do DIO mice. (**A**,**B**) Volcano plots showing the expression of 153 T cell related genes from tumors harvested at day 21 post-tumor challenge in (**A**) therapy-treated OB-RES versus OB-RES no therapy controls and (**B**) therapy-treated DIO versus DIO no therapy controls (both panels, *n* = 3–8/group). Lines represent exploratory unadjusted p-value thresholds to screen for differentially expressed target genes. (**C**) Selected differentially expressed genes. (**D**) Differentially expressed T cell related pathways queried using Reactome biological processes. NT: no therapy, TX: AdT/CpG+αCTLA-4.

We had shown previously that AdT/CpG therapeutic efficacy was dependent upon CD8^+^ T cells in this tumor model [32] and that our combinatorial AdT/CpG+αCTLA-4 therapy induced a robust CD8^+^ TIL response in lean mice that responded well to therapy (Figure 1C). For these reasons, we next sought to identify potential T cell related changes that were contributing to differential tumor outcomes over time in HFD-fed OB-RES versus DIO mice. To that end, we performed nanoString immunogenetic profiling of renal tumors at day 21, a point at which tumor burdens in OB-RES and DIO mice had diverged (Supplemental Figure S1A). Volcano plots were generated to show the expression of 153 T cell related genes in therapy-treated OB-RES versus OB-RES no therapy controls (Figure 3A) and therapy-treated DIO versus DIO no therapy controls (Figure 3B) from tumors harvested at day 21 post-tumor challenge. Lines represent exploratory unadjusted *p*-value thresholds to screen for differentially expressed target genes. Multiple genes that were differentially expressed in therapy-treated OB-RES versus OB-RES no therapy controls showed similar patterns in therapy-treated DIO versus DIO no therapy controls, although the magnitude of gene expression changes tended to be greater in OB-RES mice (Figure 3C). Next, differentially expressed genes for therapy-treated OB-RES and therapy-treated DIO mice were analyzed using the Reactome Database to compare biological processes between groups (Figure 3D). Doing so illustrated that the numbers of genes altered in general immune system pathways were greater in therapy-treated OB-RES mice than in DIO mice; similar differences were observed in Reactome processes related to interferon signaling, adaptive immune responses, cytokine signaling, and signaling by interleukins (Figure 3D). Collectively, our gene expression studies suggested that although both OB-RES and DIO mice showed beneficial T cell related alterations following AdT/CpG+αCTLA-4 therapy administration, the magnitude of these changes was greater in OB-RES mice at day 21, mirroring the improved control of renal tumor growth present in therapy-treated OB-RES mice at this time point.

### 3.4. Therapy-Treated OB-RES Mice Possess an Intratumoral Milieu Favoring T Cell Chemoattractants and Reductions in Pro-Inflammatory Mediators

**Figure 4:**
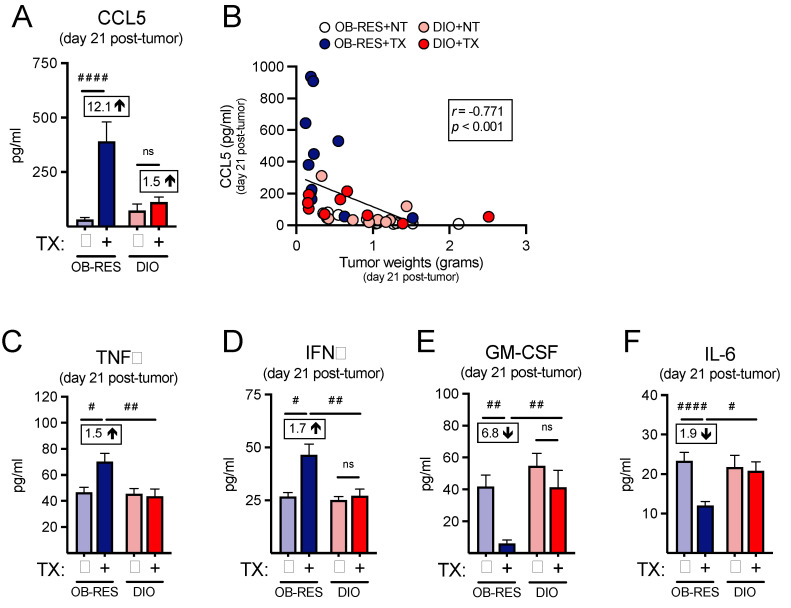
Therapy-treated OB-RES mice possess a tumor milieu favoring T cell chemoattractant signals and reductions in pro-inflammatory mediators. (**A**) The concentration of CCL5 from tumor homogenates at day 21 post-tumor challenge. (**B**) Linear regression of CCL5 concentrations versus tumor weights. The concentration of (**C**) TNFα, (**D**) IFNγ, (**E**) GM-CSF, and (**F**) IL-6 from tumor homogenates at day 21 post-tumor challenge. Data from *n* = 2 independent experiments. Fold change value calculated using no therapy control for each group. ^#^ denotes non-parametric test. ^#^ *p* < 0.05, ^##^ *p* < 0.01, ^####^ *p* < 0.0001. ns: non-significant, NT: no therapy, TX: AdT/CpG+αCTLA-4.

As we had used our nanoString profiling as a screening tool to help us identify potential changes in the intratumoral T cell responses between therapy-treated OB-RES and DIO mice, we next asked whether we could confirm our immunogenetic findings at the protein level by performing multiplex arrays, beginning with CCL5, the top differentially expressed gene within the therapy-treated OB-RES group. The intratumoral concentration of CCL5, a known T cell chemoattractant [35], was significantly increased in therapy-treated OB-RES mice (12.1-fold increase compared to OB-RES no therapy control) at day 21 relative to all other groups (Figure 4A, Kruskal–Wallis test, KW = 20.12, *p* < 0.001). CCL5 concentration correlated negatively with day 21 tumor size (Figure 4B, Spearman, *r* = −0.771, *p* < 0.0001). Therapy-treated OB-RES mice also had significant increases in the intratumoral concentrations of TNFα (Figure 4C, Kruskal–Wallis test, KW = 12.71, *p* < 0.001) and IFNγ (Figure 4D, Kruskal–Wallis test, KW = 11.22, *p* = 0.011), reflecting the robust T cell gene expression profiles detected in these mice. In contrast, intratumoral concentrations of the tumor-promoting factors GM-CSF (Figure 4E, Kruskal–Wallis test, KW = 17.95, *p* = 0.001) and IL-6 (Figure 4F, Kruskal–Wallis test, KW = 13.60, *p* = 0.004) were significantly reduced in therapy-treated OB-RES mice at day 21 post-tumor challenge. Collectively, these results illustrate that in response to AdT/CpG+αCTLA-4 therapy OB-RES mice display increased local expression of the T cell chemoattractant CCL5 and concomitant decreases in pro-tumorigenic GM-CSF and IL-6, all of which are lacking in DIO therapy-treated counterparts.

### 3.5. Therapy-Treated OB-RES Mice Have an Enhanced CD44^+^CD4^+^ TIL Response

**Figure 5:**
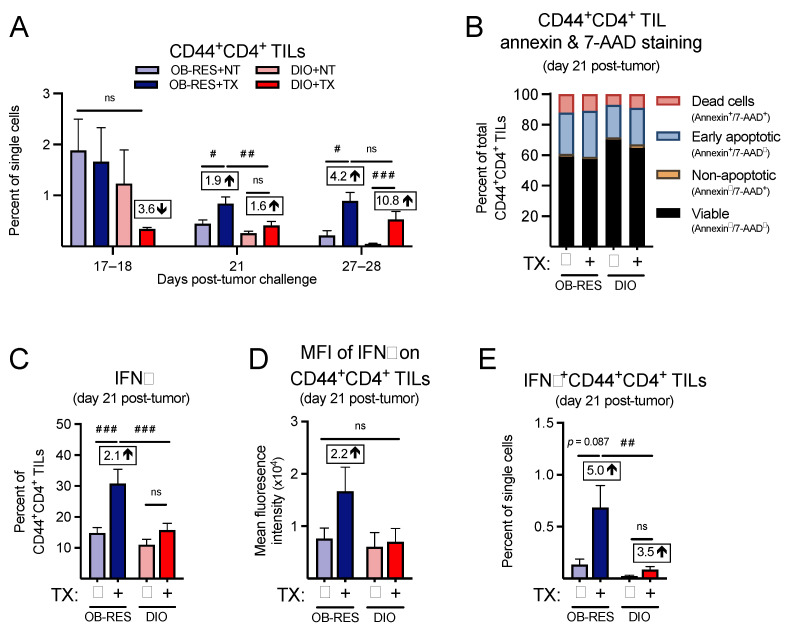
Therapy-treated OB-RES mice have a robust CD4^+^ TIL effector response that is lacking in DIO mice. (**A**) CD44^+^CD4^+^ TILs at day 17–18, day 21, and day 27–28 post-tumor challenge across groups. (**B**) Cell viability and death markers at day 21 post-tumor challenge in CD44^+^CD4^+^ TILs. (**C**) Intracellular expression of IFNγ and (**D**) the mean fluorescent intensity of IFNγ in ex vivo stimulated CD44^+^CD4^+^ TILs at day 21 post-tumor challenge. (**E**) IFNγ^+^CD44^+^CD4^+^ TILs as a percent of single cells at day 21 post-tumor challenge. Data from *n* = 2–5 independent experiments. Fold change value calculated using no therapy control for each group. ^#^ denotes non-parametric test. ^#^ *p* < 0.05, ^##^ *p* < 0.01, ^###^ *p* < 0.001. ns: non-significant, NT: no therapy, TX: AdT/CpG+αCTLA-4.

We next asked whether we could confirm our immunogenetic findings at the cellular level by performing multi-parameter flow cytometry. First, focusing on the CD4^+^ T cell response, we found that CD44^+^CD4^+^ TILs (Figure 5A) were not significantly different between groups at day 17–18 (KW = 2.80, *p* = 0.424); however, significant differences between groups emerged at day 21 (KW = 16.64, *p* < 0.001) and day 27–28 (KW = 19.17, *p* < 0.001). Post hoc tests revealed that CD44^+^CD4^+^ TILs from therapy-treated OB-RES mice were significantly elevated compared to no therapy controls and therapy-treated DIO mice at day 21. Both OB-RES and DIO therapy-treated mice displayed a significant increase in CD44^+^CD4^+^ TILs at day 27–28 compared to no therapy controls, likely due to tumor-induced reductions in the CD44^+^CD4^+^ TIL population that occurred in the absence of therapy. We next asked whether obesity was increasing the rates of cell death in CD44^+^CD4^+^ TILs from DIO mice, again focusing on the day 21 time point. However, we found only minimal changes in annexin and 7-AAD staining in CD44^+^CD4^+^ TILs (Figure 5B), suggesting that increased cell death was not a contributing factor in the divergent frequencies of CD4^+^ T cells in tumors from therapy-treated OB-RES versus DIO mice. We then examined the functional capacity of CD44^+^CD4^+^ TILs, to determine whether obesity blunted their effector function in therapy-treated mice. Therapy-treated OB-RES mice displayed a significant increase in the percentage of CD44^+^CD4^+^ TILs that expressed IFNγ^+^ relative to no therapy controls (Figure 5C, Kruskal–Wallis test, KW = 19.27, *p* < 0.001), as well as a 2.2-fold increase in the mean fluorescent intensity of IFNγ in CD44^+^CD4^+^ TILs (Figure 5D, Kruskal–Wallis test, KW = 3.03, *p* = 0.387). Neither of these changes were present in therapy-treated DIO mice. Therapy-treated OB-RES mice also had a significant increase in the overall abundance of IFNγ^+^CD44^+^CD4^+^ TILs compared to OB-RES and DIO NT controls and therapy-treated DIO mice (Figure 5E, Kruskal–Wallis test, KW = 16.89, *p* = 0.001). Thus, therapy-treated DIO mice mounted a delayed effector CD4^+^ TIL at days 27–28 but displayed reduced frequencies and function in effector CD4^+^ TILs at day 21 compared to their therapy-treated OB-RES counterparts.

### 3.6. Therapy-Treated OB-RES Mice Have a Sustained CD44^+^CD8^+^ TIL Response over Time

**Figure 6:**
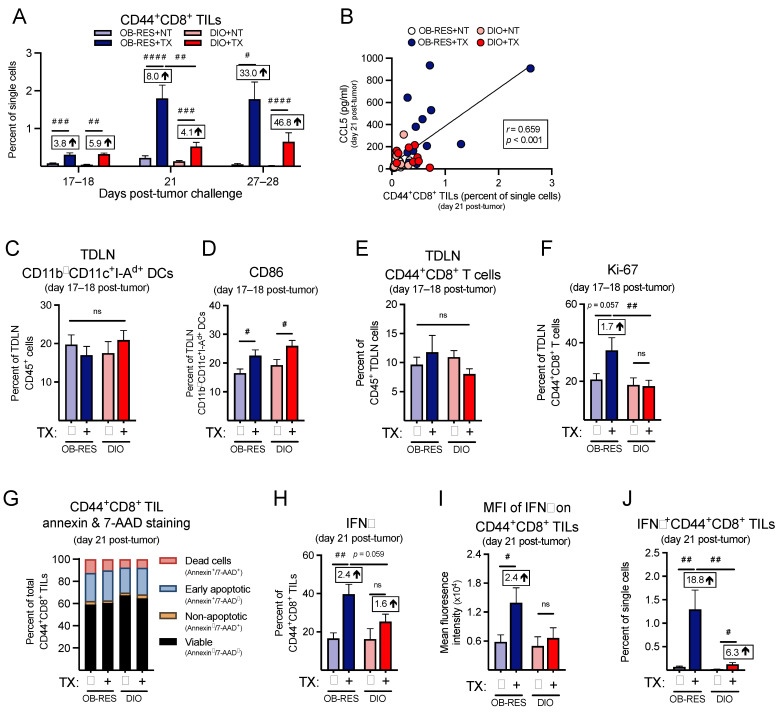
Therapy-treated OB-RES mice have a sustained CD8^+^ TIL effector response that is blunted in DIO mice. (**A**) CD44^+^CD8^+^ TILs at day 17–18, day 21, and day 27–28 post-tumor challenge across groups. (**B**) Linear regression of CCL5 concentrations versus CD44^+^CD8^+^ TILs at day 21 post-tumor challenge. (**C**) The frequencies of CD11b^—^CD11c^+^I-A^d+^ TDLN DCs and (**D**) CD86 expression on TDLN CD11b^—^CD11c^+^I-A^d+^ DCs; (**E**) the frequencies of TDLN CD44^+^CD8^+^ T cells and (**F**) the proliferative capacity (Ki-67^+^) in TDLN CD44^+^CD8^+^ T cells at days 17–18 post-tumor challenge. (**G**) Cell viability and death markers at day 21 post-tumor challenge in CD44^+^CD8^+^ TILs. (**H**) Intracellular expression of IFNγ and (**I**) the mean fluorescent intensity of IFNγ in ex vivo stimulated CD44^+^CD8^+^ TILs at day 21 post-tumor challenge. (**J**) IFNγ^+^CD44^+^CD8^+^ TILs as a percent of single cells at day 21 post-tumor challenge. Data from *n* = 2–5 independent experiments. Fold change value calculated using no therapy control for each group. ^#^ denotes non-parametric test. ^#^ *p* < 0.05, ^##^ *p* < 0.01, ^###^ *p* < 0.001, ^####^ *p* < 0.0001. ns: non-significant, NT: no therapy, TX: AdT/CpG+αCTLA-4.

Next, focusing on the CD8^+^ T cell response, we found that the frequencies of CD44^+^CD8^+^ TILs (Figure 6A) were significantly different between groups at day 17–18 (KW = 24.78, *p* < 0.001), day 21 (KW = 54.95, *p* < 0.001), and day 27–28 (KW = 24.98, *p* < 0.001), with CD44^+^CD8^+^ TILs peaking at day 21 for OB-RES therapy-treated mice. Post hoc tests revealed that therapy-treated mice displayed significant increases in CD44^+^CD8^+^ TILs compared to no therapy controls at all time points examined; however, CD44^+^CD8^+^ TILs from therapy-treated OB-RES mice were significantly elevated compared to therapy-treated DIO mice at day 21 and OB-RES mice were able to sustain these CD44^+^CD8^+^ TIL frequencies through days 27–28. Although therapy increased the relative frequencies of CD44^+^CD8^+^ TILs in DIO mice versus no therapy controls, the magnitude of this response was blunted relative to that seen in therapy-treated OB-RES mice (Figure 6A). CCL5 concentration correlated positively with CD44^+^CD8^+^ TIL frequencies across all groups (Figure 6B, Spearman, *r* = 0.659, *p* < 0.001).

Because tumor antigen-specific T cell responses originate in TDLNs [36], we examined immune responses here at an earlier day 17–18 time point, to determine whether deficiencies existed in therapy-treated DIO animals relative to OB-RES mice that might contribute to the divergent effector T cell responses detected within renal tumors at day 21. We found no differences in the abundance of CD11b^—^ dendritic cells (DCs) in TDLNs harvested at days 17–18 post-tumor challenge (Figure 6C, Kruskal–Wallis test, KW = 1.96, *p* = 0.582); although therapy did increase CD86 expression on DCs from both OB-RES and DIO mice (Figure 6D, Kruskal–Wallis test, KW = 11.50, *p* = 0.009). Furthermore, no differences were observed in the abundance of TDLN CD44^+^CD8^+^ T cells (Figure 6E, Kruskal–Wallis test, KW = 3.19, *p* = 0.364) or their activation status, as measured by CD69 staining (Supplemental Figure S2A, Kruskal–Wallis test, KW = 0.94, *p* = 0.816). Within group differences were not observed for CTLA-4 expression on TDLN CD44^+^CD8^+^ T cells (Supplemental Figure S2B, Kruskal–Wallis test, KW = 5.80, *p* = 0.122); however, CD44^+^CD8^+^ T cells from therapy-treated OB-RES mice displayed a significant increase in CTLA-4 expression compared to T cells from therapy-treated DIO mice. Despite their increased CTLA-4 expression, TDLN CD44^+^CD8^+^ T cells from therapy-treated OB-RES mice possessed a heightened proliferative capacity as evaluated by Ki-67 staining, relative to TDLN CD44^+^CD8^+^ T cells from DIO therapy-treated mice (Figure 6F, Kruskal–Wallis test, KW = 8.33, *p* = 0.040).

We next examined T cell expression of chemokine receptors, to determine whether host obesity was associated with reductions that might further explain the weakened T cell responses detected in therapy-treated DIO mice at day 21. We began by examining expression of CXCR3, a receptor for CXCL9, CXCL10, and CXCL11, all chemokines that regulate effector CD8^+^ T cell trafficking [37]. However, we found no differences across groups for CXCR3 expression on CD44^+^CD8^+^ TILs (Supplemental Figure S3A, Kruskal–Wallis test, KW = 4.30, *p* = 0.231). We did find therapy-induced reductions in CCR4 (Supplemental Figure S3B, Kruskal–Wallis test, KW = 24.73, *p* < 0.001) and CCR5 (Supplemental Figure S3C, Kruskal–Wallis test, KW = 13.73, *p* = 0.003), both of which are receptors for the T cell chemoattractants CCL4 and CCL5 [38], but these reductions were comparable on T cells from both OB-RES and DIO mice.

We next asked whether obesity was increasing the rates of cell death in TILs from DIO mice, again focusing on the day 21 time point. However, we found only minimal changes in annexin and 7-AAD staining in CD44^+^CD8^+^ TILs (Figure 6G), suggesting that increased cell death was not a contributing factor in the divergent frequencies of CD8^+^ T cells in tumors from therapy-treated OB-RES versus DIO mice. We then examined the functional capacity of CD44^+^CD8^+^ TILs, to determine whether obesity blunted their effector function in therapy-treated mice. We found that both the intracellular expression of IFNγ (Figure 6H, Kruskal–Wallis test, KW = 15.22, *p* = 0.002) and the mean fluorescent intensity of IFNγ (Figure 6I, Kruskal–Wallis test, KW = 6.88, *p* = 0.030) in stimulated CD44^+^CD8^+^ TILs were significantly increased in therapy-treated OB-RES mice relative to OB-RES NT mice at day 21 post-tumor challenge. In contrast, no significant increases in IFNγ expression were present in therapy-treated DIO mice relative to their DIO NT controls (Figure 6H, *p* = 0.084 and Figure 6I, *p* = 0.307). Therapy-treated OB-RES mice also had a significant increase in the abundance of IFNγ^+^CD44^+^CD8^+^ TILs compared to OB-RES and DIO no therapy controls and therapy-treated DIO mice (Figure 6J, Kruskal–Wallis test, KW = 25.42, *p* < 0.001).

Finally, we asked whether the reduced frequency of IFNγ^+^CD44^+^CD8^+^ TILs in therapy-treated DIO mice was accompanied by increases in the frequencies of myeloid-lineage cells within tumor-bearing kidneys. We found no differences across groups in CD11b^+^ (Supplemental Figure S3D, Kruskal–Wallis test, KW = 0.26, *p* = 0.968) or CD11b^—^ (Supplemental Figure S3E, Kruskal–Wallis test, KW = 3.46, *p* = 0.326) DC subsets. Both OB-RES and DIO therapy-treated mice had significant reductions in total MDSCs (Supplemental Figure S3F, Kruskal–Wallis test, KW = 17.66, *p* = 0.001) and enhanced CD44^+^CD8^+^ T cell to MDSC ratios (Supplemental Figure S3G, Kruskal–Wallis test, KW = 36.26, *p* < 0.001). However, the effect size was again much larger in therapy-treated OB-RES mice, which showed a 134-fold increase in the CD44^+^CD8^+^ T cell to MDSC ratio relative to OB-RES NT controls, versus a 58-fold increase in DIO treated versus NT mice (Supplemental Figure S3G). Collectively, these results illustrate that in response to AdT/CpG+αCTLA-4 therapy OB-RES mice display heightened and sustained CD8^+^ TIL responses that are associated with increased local expression of the T cell chemoattractant CCL5 and concomitant decreases in pro-tumorigenic GM-CSF, IL-6, and myeloid-derived suppressor cells, all of which are lacking in DIO therapy-treated counterparts.

## 4. Discussion

Here, we examined the effects of obesity on T cell responses following the administration of a novel combinatorial immunotherapy comprised of in situ T cell priming plus αCTLA-4 in mice with established, orthotopic renal tumors. Foremost, we found that this therapeutic strategy is highly efficacious in both chow-fed lean mice and HFD-fed OB-RES mice but has reduced efficacy in HFD-fed DIO animals, resulting in progressive tumor outgrowth at later time points. Diminished therapeutic efficacy in DIO mice was characterized by multi-factorial defects in effector CD4^+^ and CD8^+^ T cell responses, which included reduced intratumoral concentrations of the T cell chemoattractant CCL5, heightened concentrations of the pro-tumorigenic GM-CSF, reduced functionality in both CD44^+^CD8^+^ and CD44^+^CD4^+^ TILs, and an unfavorable CD44^+^CD8^+^ T cell to MDSC ratio. Obesity-associated T cell deficiencies extended beyond the tumor microenvironment, as evidenced by reduced proliferative capacity of CD44^+^CD8^+^ T cells in tumor-draining lymph nodes. The net result was progressive tumor outgrowth in therapy-treated DIO mice. In addition, protective immune changes in therapy-treated OB-RES mice were typically of greater magnitude and/or duration, relative to those seen in therapy-treated DIO mice. Our results suggest that immunotherapy resistance in the context of host obesity is caused by blunted effector CD4^+^ and CD8^+^ T cell responses. Thus, finding ways to magnify and sustain the number and function of effector T cells within renal tumors from individuals with obesity may be critical for achieving improved outcomes after immunotherapy administration in patients who fail to respond to therapy.

Clinically, the association between obesity and the efficacy of ICB agents remains uncertain in the context of advanced RCC [26]. Subgroup analysis within a recent meta-analysis showed that RCC patients with a high BMI undergoing ICB tended to experience worse outcomes (HR = 1.10, CI: 0.50–2.41, *p* = 0.810) [27]. This trend is supported by our recent analysis of outcomes in RCC patients following ICB administration, wherein patients with obesity had worse progression-free survival and overall survival [9]. In contrast, Sanchez et al. [28] found no significant association between obesity and ICB outcomes in RCC but these authors did identify a strong trend toward beneficial effects of obesity. Therefore, the full effects of obesity on ICB-induced immune responses and clinical outcomes in patients with RCC remains unclear. This fact emphasizes the need for continued preclinical modeling of co-morbidities, such as obesity. However, preclinical obesity modeling often overlooks diet effects with HFD groups being compared to chow-fed, lean controls [20]. Recent modeling from our laboratory demonstrates that energy intake and diet composition matching can be achieved in BALB/c mice with long-term HFD-feeding resulting in subsets of mice that are either OB-RES or DIO [31]. Importantly, diet-matched OB-RES mice share a comparable metabolic phenotype (e.g., adipose levels, serum leptin, insulin response) to chow-fed, lean controls, whereas DIO mice display increased adiposity and elevated serum concentrations of pro-inflammatory mediators [31]. Therefore, in the present study we were able to assess the efficacy of combinatorial therapy and T cell responses over time in OB-RES versus DIO renal tumor-bearing animals independent of potentially confounding diet effects.

Mounting evidence indicates that obesity negatively impacts immune function and immune-based interventions, with multiple laboratories, including our own, reporting that increased adiposity blunts anti-tumor immunity and increases tumor growth in multiple preclinical models [3,4,5,6,7,8,9,10,11,12]. Previously, we determined that DIO was associated with increased intratumoral frequencies of immunosuppressive dendritic cells (DC) [3] and myeloid-derived suppressor cells (MDSCs) [4] and a reduced efficacy of AdT/CpG in Renca tumor-bearing mice [3]. More recently, we found that DIO diminished the efficacy of αPD-1-based immunotherapy in orthotopically injected Renca tumor-bearing mice, with therapy non-responders displaying an unfavorable ratio of activated CD8^+^ T cell to MDSCs within tumors [9]. Additional studies have suggested that obesity dysregulates MDSCs [8,39], thereby promoting an immunosuppressive tumor microenvironment. A wide variety of obesity-induced mechanistic changes have been reported. These include impairments in natural killer (NK) cell function [40,41], increased production of pro-inflammatory immunosuppressive cytokines [6], and reduced effector function of CD8^+^ T cell infiltration into tumors [12,42].

Our current examination of combinatorial therapy in diet-matched OB-RES and DIO mice provides additional insight on obesity-induced dysregulation of anti-tumor immune mechanisms. In OB-RES mice, immunotherapy induced a robust remodeling of immune responses within the renal tumor microenvironment, characterized by increased concentrations of T cell chemoattractants such as CCL5 and decreased concentrations of pro-tumorigenic factors such as GM-CSF and IL-6. This type of dramatic intratumoral immune remodeling was greatly reduced in mice with obesity, resulting in a waning immune response and tumor escape. The ability of chemokines, cytokines, and growth factors and/or their receptors to serve as a prognostic markers in cancer progression is under investigation [43] and targeting those markers to enhance anti-tumor immunity and/or blunt tumor growth is emerging as a potential therapeutic approach [44,45,46]. Previous data from our laboratory demonstrate that DIO mice with mammary carcinoma display reduced immunotherapeutic efficacy, in part due to CXCL1-driven accumulation of FASL^+^ MDSCs inducing apoptosis in effector CD8^+^ TILs [10]. Furthermore, poor responses to a PD-1-based combinatorial immunotherapy in orthotopically injected Renca tumor-bearing mice was associated with elevated intratumoral IL-1β [9]. Our current data suggest that boosting intratumoral anti-tumor effector signals, such as CCL5, or blocking pro-inflammatory signals such as GM-CSF and IL-6, could serve as therapeutic options in combination with ICB, particularly in patients with combined obesity and RCC. Interestingly, when comparing the effects of DIO on the efficacy of αPD-1-based [9] versus αCTLA-4-based immunotherapy, it appears that DIO mice are more responsive to our αCTLA-4-based immunotherapy strategy. However, both combinatorial therapies fail to induce a significant reduction in renal tumor weight compared to their therapy-treated OB-RES counterparts, suggesting that obesity impairs the response to both combinatorial therapy strategies. Future studies are needed to further explore the differential effects of obesity on therapeutic efficacy and determine whether therapeutic response can be bolstered by the dual administration of αPD-1 and αCTLA-4.

The obesity-induced changes we identified at the molecular level were reinforced by our cellular analyses of the tumor microenvironment and tumor-draining lymph nodes. Obesity impaired the ability of both CD4^+^ and CD8^+^ TILs to mount productive and sustained effector responses. While the anti-tumor activity of CD8^+^ T cells is well documented [47], emerging literature details essential anti-tumor activities of CD4^+^ T cells, including their ability to generate tumor-specific cytolytic responses and mediate the anti-tumor response through the production of cytokines [48,49,50]. The majority of publications detailing the impact of obesity on T cells have focused on the CD8^+^ subset [13], but here we are reporting an obesity-induced reduction in both CD4^+^ and CD8^+^ T cell effector responses. Obesity-induced factors altered T cell dynamics, reducing their ability to sustain a functional presence within the tumor microenvironment over time in therapy-treated mice. The reduced intratumoral effector function and deficiency in upstream proliferative capacity within the tumor-draining lymph node coincides with subsequent tumor escape. Obesity did not induce greater cell death within T cell subsets but did diminish CD8^+^ T cell proliferation downstream of TCR engagement, providing another contributing factor to the blunted CD8^+^ TIL expansion seen in therapy-treated DIO mice. A recent study by Ringel et al. [12] found that in tumor models wherein HFD-induced accelerated outgrowth, rapid tumor growth was associated with a reduced number, proliferative capacity, and anti-tumor activity of CD8^+^ TILs in therapy naive mice. Ringel et al. [12] failed to observe an effect of HFD on CD8^+^ TIL response in the Renca model when injected subcutaneously, a similar finding when comparing our orthotopically injected no therapy DIO mice to no therapy OB-RES controls. The use of our immunostimulatory therapy in orthotopically injected mice likely alters the immunogenic profile and susceptibility of T cells to the adverse effects of chronic HFD in the Renca model resulting in an inability to mount a productive effector T cell response and reduced therapeutic efficacy.

Lastly, obesity induced an unfavorable activated CD8^+^ T cell to MDSC ratio within the tumor microenvironment. While both OB-RES and DIO therapy-treated mice had significant reductions in total MDSCs and enhanced CD44^+^CD8^+^ T cell to total MDSC ratios, the magnitude of these changes in therapy-treated OB-RES mice (134-fold increase) far exceeded that seen in therapy-treated DIO mice (58-fold increase). A less favorable CD44^+^CD8^+^ T cell to total MDSC ratio within the tumor microenvironment may contribute to the impairment in CD8^+^ T cell effector function we observed within the tumor microenvironment. We found no differences across groups in intratumoral CD11b^+^ or CD11b^—^ DC subsets, or the ability of tumor-draining lymph node DCs to express CD86 in response to therapy. Examining chemokine receptor expression, we observed no differences across groups for CXCR3 expression on CD44^+^CD8^+^ T cells, but found therapy-induced reductions in CCR4 and CCR5, possibly due to clathrin-mediated endocytosis or lipid rafts/caveolae-dependent internalization [51]. Therefore, obesity-induced alterations in intratumoral T cell abundance and function were related to intrinsic changes in T cell activation and priming, as well as an unfavorable shift away from T cell chemoattractant signals and toward pro-inflammatory signals within the tumor microenvironment. Collectively, these changes likely contributing to the observed blunting of CD8^+^ TIL expansion over time and uncontrolled tumor growth.

Our current study contains several limitations. The Renca model is associated with rapid and aggressive tumor growth and future studies could characterize the effects of DIO on metastatic progression following resection of primary renal tumors, to better mimic clinical parameters. Furthermore, the pleiotropic effects of chronic HFD makes it difficult to pinpoint a singular mechanism by which obesity diminishes T cell responses to immunotherapy administration in renal tumors from DIO mice. Additionally, our findings do not rule out deficiencies in other immune cells and additional exploration of obesity-induced alterations driving the production of chemokines and cytokines from innate immune cells is warranted. With mounting evidence suggesting that modifiable lifestyle factors, such as diet and elevated adiposity, can impact therapeutic outcomes [20,21,22,23,24], future studies are needed to investigate if dietary or pharmacological intervention strategies can blunt obesity-induced alterations to T cell populations to improve combinatorial therapy efficacy. Recent studies from our laboratory have investigated the effects of time-restricted feeding and the glucose-limiting drug, acarbose, on Renca tumor growth and response to therapy. Time-restricted feeding differentially impacted tumor growth with older, HFD-fed mice experiencing reduced renal tumor bioluminescence; however, time-restricted feeding did not alter tumor weights or intratumoral immune responses and failed to improve anti-CTLA-4 monotherapy [52]. Acarbose delayed renal tumor growth in a CD8^+^ T cell-dependent manner, increasing the abundance and maintaining the effector function CD8^+^ TILs [53]. When combined with αPD-1, acarbose significantly reduced lung metastasis, suggesting that combining acarbose with current therapies may improve outcomes [53]. Lastly, it would be of interest to determine whether weight loss could reverse the obesity-induced immunological deficiencies and improve response to combinatorial immunotherapy. Here, our results illustrate that combinatorial therapy is able to amplify and sustain protective immune responses and enhance tumor clearance in HFD-matched, OB-RES mice; however, this combinatorial therapy was unable to sustain prolonged protective immunity in DIO mice resulting in progressive tumor growth over time. Future studies are needed to better characterize obesity-induced disturbances in T cell priming, as well as obesity-induced alterations in other cell types important in immunosurveillance and anti-tumor immunity.

## 5. Conclusions

Our finding that αCTLA-4 fails as a monotherapy in lean mice with established renal tumors mirrors the clinical setting, wherein αCTLA-4 is FDA approved for combinatorial use in conjunction with αPD-1 but not as a monotherapy in patients with advanced renal cancer [17]. Our results suggest that αCTLA-4 may be effective in lean renal cancer patients if combined with an upstream in situ T cell-priming agent, such as the AdT/CpG approach we used here. This type of combinatorial strategy may also avoid some of the immune-related adverse events observed after co-administration of αCTLA-4 plus αPD-1. Additionally, our results contribute several important findings to the study of obesity as a modifier of anti-tumor immunity and cancer immunotherapy outcomes since our preclinical cancer modeling more closely reflected the aging, and frequently overweight, population of cancer patients who receive therapy [54]. CD4^+^ T cells are critical for sustaining CD8^+^ cytotoxic T cell immunity [16], and our results here suggest that closer attention should be paid to CD4^+^ T cell immune responses following immunotherapy administration, particularly in the context of obesity. Thus, our study has not only revealed a novel combinatorial approach for αCTLA-4 use in renal cancer but has contributed a deeper understanding of the likely myriad ways in which host obesity influences anti-tumor immunity and immunotherapy outcomes. Additional investigation of our findings should be conducted in other preclinical tumor models and cancer patients to determine the extent of their applicability.

## Data Availability

Gene expression data from this study are available from the corresponding author on reasonable request.

## Supplementary Materials

The following are available online at https://www.mdpi.com/article/10.3390/cancers13102295/s1, Table S1: List of T cell related genes from Figure 3; Figure S1: Therapy-treated obese-resistant (OB-RES) mice have a sustained reduction in tumor growth over time, whereas therapy-treated diet-induced obese (DIO) mice experience an acute response followed by tumor outgrowth; Figure S2: Expression of T cell markers in activated CD8+ and CD4+ tumor-draining lymph node (TDLN) T cell at days 17–18 post-tumor challenge in OB-RES versus DIO mice; Figure S3: Therapy-treated OB-RES mice have reduced myeloid-derived suppressor cells (MDSCs) and improved CD44^+^CD8^+^ TIL to MDSC ratio at day 21 post-tumor challenge.
