# Supplementary figures and images for: Diet-Induced Obesity Impairs Outcomes and Induces Multi-Factorial Deficiencies in Effector T Cell Responses Following Anti-CTLA-4 Combinatorial Immunotherapy in Renal Tumor-Bearing Mice
Source: Cancers (Basel). 2021 May 11;13(10):2295. doi: 10.3390/cancers13102295 (PMC8151089; doi:10.3390/cancers13102295)

A

## Renal tumor weights

OB-RES+NT    DIO+NT  
OB-RES+TX    DIO+TX

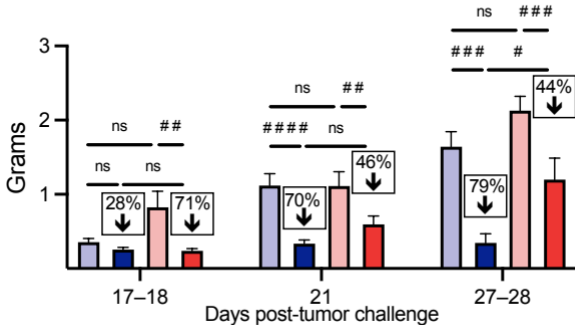

Supplement: Supplementary file 1 [file cancers-13-02295-s001.zip › Supplemental Figure 1.pdf]

A

CD69

(day 17–18 post-tumor)

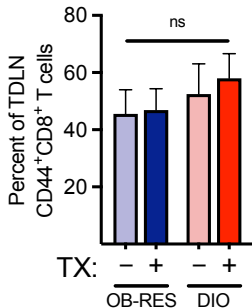

B

CTLA-4

(day 17–18 post-tumor)

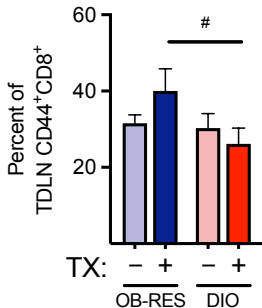

Supplement: Supplementary file 1 [file cancers-13-02295-s001.zip › Supplemental Figure 2.pdf]

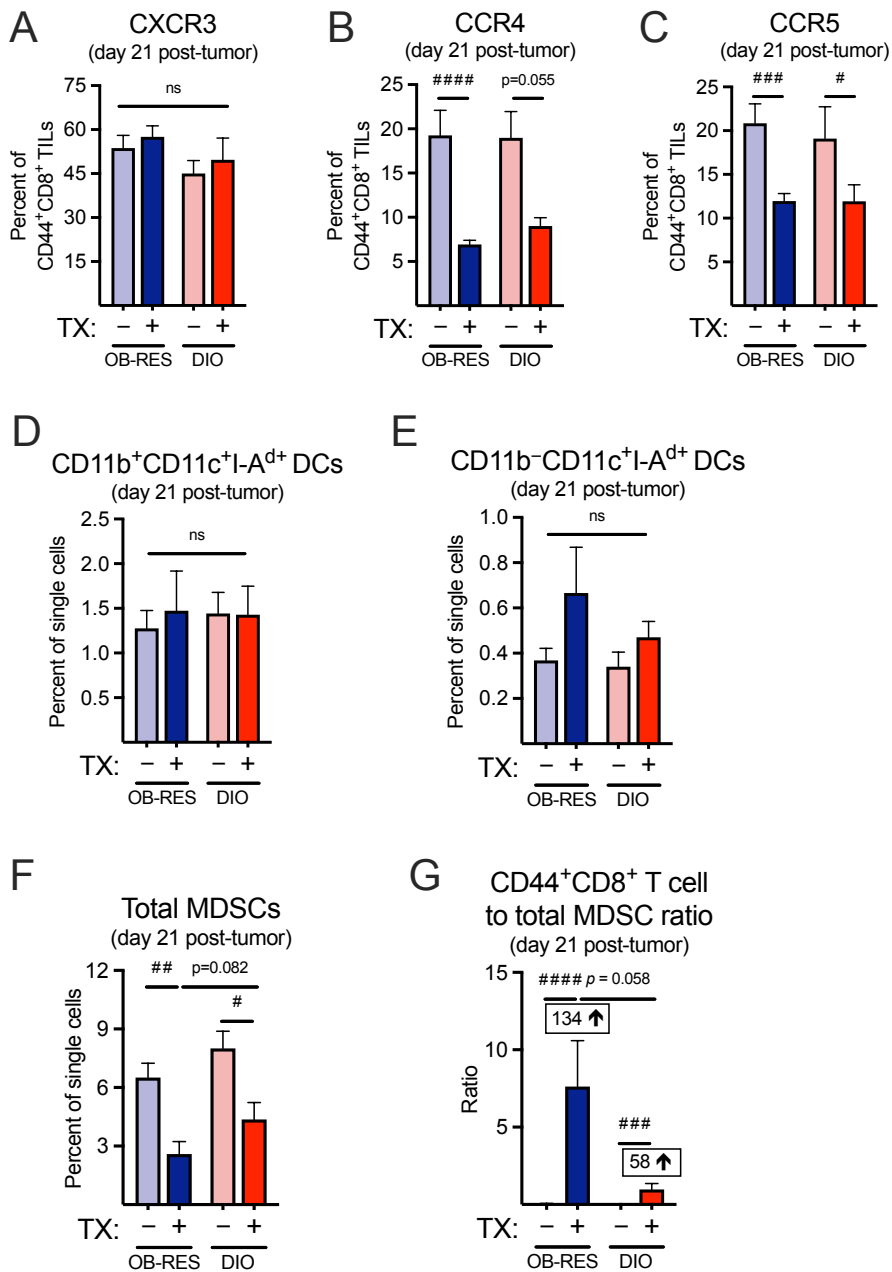

Supplement: Supplementary file 1 [file cancers-13-02295-s001.zip › Supplemental Figure 3.pdf]
